# Supplementary material for: Mental health and risk of death and hospitalization in COVID–19 patients. Results from a large-scale population-based study in Spain
Source: PLoS One. 2024 Feb 12;19(2):e0298195. doi: 10.1371/journal.pone.0298195 (PMC10861053; doi:10.1371/journal.pone.0298195)
Supplement: Attachment — Submitted filename: Peer Review_PLOSOne_COVID.docx [file pone.0298195.s001.docx]

Peer Review

Mental health and risk of death and hospitalization in COVID-19 patients. Results from a large-scale population-based study in Spain.

Line 62: Please clarify what you mean by “risk factors”. Risk factors for what?

Line 64: You say “Also, socioeconomic status [3,6-7], education level [6] and race have been associated as risk factors for severity and mortality…”. Please indicate what is meant by severity. Symptom severity? Infection severity? Length of symptoms?

Lines 66 and 68 and throughout: The term “mental disorders” can be stigmatizing. I recommend that this be changed to “mental health conditions” or “mental health concerns” or “mental health diagnoses”.

Lines 74-75: You indicate that a number of studies have been done on the impact of mental health on COVID-19 outcomes and vice versa, but do not include citations. Please add them in.

Lines 77-84: Please revisit this text for clarity. I suggest breaking this up into multiple sentences.

Introduction: The paper feels a bit imbalanced. There are approximately 2 pages of introductory text and approximately 6 pages of discussion text. The discussion reviews previous findings, but should more clearly contextualize the findings from the current study within previous literature. The introduction should more clearly state the rationale for the study (some of this could be -pulled from the discussion) and what gap this study addresses. I note the use of the term “explore” in line 85, but I would hope that the researchers had a specific hypothesis/hypotheses that could be clearly stated, especially as they use the term “evaluate” on line 86. While the authors present interesting results, without a hypothesis/hypotheses and clearly stating the rationale/gap in the literature, it is not clear what this study adds/why it is important.

Lines 97-100: For replication purposes as well as evaluation of systematic missingness, do patients have the option of opting out of providing their data for use in studies? If so, please describe. If not, please indicate.

Lines 94, and 101-103 included acronyms that may be unfamiliar to readers. Please expand these and any others in the text upon first use (e.g., PRECOVID, IACS, CEICA, etc.).

Line 106: Typo of the word “enrollment”

Line 109: What is meant by “Follow-up ended invariably on 22 August 2021”. Why? Please explain. Was this a pre-determined date?

Lines 114-115: You collapsed ethnicity into a dichotomous (native v. migrant) variable. Please include numbers regarding the ethnic groups represented in the study (*n*s, percentages) in the text of the methods. As migration status may be related to the variables of interest at varying degrees per ethnic group (depending on country of origin, racial tensions, cultural traditions regarding accessing healthcare and/or number of people in household and/or how mental health conditions are viewed, health status mandates for migration, etc.), sensitivity analyses should also be done per ethnic group.

Lines 116-117, superscript d in Table 1: For replication and interpretation purposes, please cite and clearly define the deprivation index utilized in this study.

Line 127: Please articulate which diagnostic labels were renamed to facilitate clinical interpretation.

Lines 146-148: What was your reasoning for conducting all analyses by gender, as opposed to the other variables that you mentioned have previously been indicated as risk factors for poor COVID-19 outcomes (lines 62-65), or the variables with significant p-values in Table 1? If this was pre-determined (and it sounds like it was), please include this rationale in the introduction section.

Lines 146-147: The phrase, “..examine the likelihood of 30-day all-cause mortality and COVID-19 related hospitalization (dependent variable…” makes it sound like you created some sort of composite of mortality and COVID-19 hospitalization and ran one regression. Please specify that you are describing different models. You should also somewhere in this section (recommended) or elsewhere describe why it is important to look at both COVID-19 hospitalization AND all-cause mortality when drawing conclusions. Please describe how this comparison lends critical information for testing your hypotheses.

Line 150: How did you determine which covariates to include in the models?

Statistical Analysis section: How were missing data handled?

Table 1: I appreciate the inclusion of the percent representation for the migrant group within the overall category of individuals with schizophrenia. Please include additional clarification on the percent representation specifically within the migrant population. For instance, could you provide the percent of individuals with schizophrenia among migrants, based on the total number of migrants? This clarification would enhance the interpretation of the data and contribute to a more nuanced understanding of the prevalence within the migrant subgroup. I have the same comment for the other characteristics listed in Table 1.

Lines 176-177: Please also include the *n*s and percentages for those without mental health diagnoses and COVID-19.

Line 197: Please double check this sentence. Did you mean that “None of the mental health diagnoses studied affected the likelihood of COVID-19-related hospitalization in men.”?

Lines 228-231: I appreciate that you have described some of particulars related to migrant status as related to the “healthy migrant effect”. Please also provide alternate explanations for the migrant findings.

Discussion: For men, living in most deprived areas decreased mortality risk (14.5%) but living in a less deprived area increased hospitalization risk (11%). And being a migrant increased hospitalization risk by 74%. For women, being a migrant decreased mortality risk substantially (nearly 50%), but increased hospitalization risk by 90%. And living in a less deprived area increased hospitalization risk by 12%. These findings and potential implications need to be expounded on in the discussion.

Lines 246-260: Thank you for detailing some of the previous work on schizophrenia and COVID-19 mortality and related hospitalization. I recommend that you include a sentence or two detailing what your study adds to this literature.

Lines 261-292: From this literature, I am left questioning why you did not include variables related to alcohol and tobacco use, as well as caregiver/family support, and hygiene practices in this study. Please seriously consider including these and if it is not feasible, please describe why and how this may limit the interpretation of the results. I see that you have done so for alcohol and tobacco use and BMI (lines 329-334), but please include other limitations related to lack of inclusion of variables related to caregiver/family support, and hygiene practices.

Lines 293-296: This sentence, “As we saw, in some cases, multimorbidity….more common in patients with SMI” made me question why you did not delineate specific chronic diseases in this study. I appreciate your reliance on other literature; however, if you want to make this claim (as well as the claim in lines 318-320), I would like to see these analyses broken down by chronic disease type.

Lines 336-339: Great point regarding the COVID-19 pandemic waves. I also wonder about lockdown/quarantine procedures/recommendations in Aragon, Spain. Please include these details somewhere in the text. This will help with reader understanding of how these results may generalize (OR NOT) to other populations.
